# Multiple Analyses Reveal Evidence for Three New Species of *Collybia* (Clitocybaceae, Basidiomycete) from China

**DOI:** 10.3390/jof11050371

**Published:** 2025-05-13

**Authors:** Yue Qi, Aiguo Xu, Liu Yang, Hongbo Guo, Yaobin Guo, Fashuang Wan, Ruiheng Yang, Ying Pei, Xiaodan Yu

**Affiliations:** 1 College of Biological Science and Technology, Shenyang Agricultural University, Shenyang 110866, China; qiyue_syau@126.com (Y.Q.); mushroom_syau@163.com (L.Y.); imguoyaobin@163.com (Y.G.); 18385286792@163.com (F.W.); 2 Alpine Fungarium, Xizang Plateau Institute of Biology, Lhasa 100101, China; sws_xag@sti.xizang.gov.cn; 3 College of Life Engineering, Shenyang Institute of Technology, Fushun 113122, China; yxd0118@163.com; 4 Institute of Edible Fungi, Shanghai Academy of Agricultural Sciences, Shanghai 201106, China; yangruiheng@126.com; 5 Shenyang Research Institute of Chemical Industry, Shenyang 110021, China; peiying@sinochem.com

**Keywords:** mushrooms, new taxa, morphological features, phylogenetic relationships

## Abstract

Three new species of *Collybia* in China, *Collybia clavipes*, *C. carnea* and *C. violea*, are originally reported and described based on morphological characteristics and molecular data. This study provides detailed morphological descriptions of these three new species of *Collybia*, which can be accurately distinguished from other species within the genus *Collybia*. Phylogenetic relationships of Clitocybaceae were analyzed using a four-loci combined dataset (ITS-nrLSU-*rpb2*-*tef1-α*), and the results show that the three newly discovered species of *Collybia* form three distinct lineages, respectively. Based on the combination of morphological and molecular methods, these three newly collected species of *Collybia* are confirmed as new to science. A theoretical basis is provided for the species diversity of *Collybia*.

## 1. Introduction

Based on phylogenetic analyses over the past two decades, many new genera have been established to accommodate traditional *Clitocybe* species [1], such as *Cleistocybe* Ammirati, A.D. Parker & Matheny [2], *Trichocybe* Vizzini [3], *Atractosporocybe* P. Alvarado, G. Moreno & Vizzini, *Leucocybe* Vizzini, P. Alvarado, G. Moreno & Consiglio and *Rhizocybe* Vizzini, G. Moreno, P. Alvarado & Consiglio [4], *Pulverulina* Matheny & K.W. Hughes 2020 [5] and *Spodocybe* Z.M. He & Zhu L. Yang [6]. However, it is noteworthy that *Clitocybe* remains polyphyletic [1,4,7,8,9,10,11,12,13]. When He et al. [14], based on phylogenetic and phylogenomic analyses, reconstructed the phylogenetic framework of Clitocybaceae and recircumscribed the genera within the family, a large number of traditional *Clitocybe* and *Lepista* species were transferred to different clades of the genus *Collybia*, and the two genera became monophyletic groups.

Species of *Collybia* have a widespread distribution all over the world. However, new taxa of *Collybia* had rarely been reported and described in China in recent years until He et al. [14], who proposed fourteen new *Collybia* species in China. In this present study, several interesting specimens of *Collybia* were collected from southwestern and northeastern China. To identify whether these specimens of *Collybia* are new to science, detailed phylogenetic and morphological examinations were conducted, resulting in possibly novel additions to the genus *Collybia*.

## 2. Materials and Methods

### 2.1. Specimens and Morphological Studies

Specimens of *Collybia* were collected from southwestern and northeastern China from 2019 to 2024. Some basidiomes were dehydrated with silica gel and used for DNA extraction; the remaining basidiomes were dehydrated with an electric drying oven at 50 °C and stored in the Fungal Herbarium of Shenyang Agricultural University (SYAU-FUNGI). Detailed information is given in Table 1. Methods for morphological observation followed Qi et al. [15]. The color codes followed the Methuen Handbook of Color [16]. For observation of the spore surface, the gills of the dried specimens were coated with gold and examined with a ZEISS Ultra Plus Scanning Electron Microscope (SEM) (Oberkochen, Germany).

### 2.2. DNA Extraction, PCR, and Sequencing

Genomic DNA was extracted from dried specimens using the cetyltrimethylammonium bromide (CTAB) method [17]. Polymerase chain reaction (PCR) was performed to amplify sequences of the ITS, nrLSU, *rpb2*, and *tef1-α* regions. Primers ITS1 and ITS4 [18] were used for the ITS region; primers LROR and LR5 [19] were used for the nrLSU region; primers bRPB2-6F and bRPB2-7.1R [20] were used for the *rpb2* region; and primers EF1-983F and EF1-1953R [21] were used for the *tef1-α* region. The PCR procedure was performed following Qi et al. [15].

### 2.3. Phylogenetic Analyses

Based on the latest phylogenetic studies on Clitocybaceae [14], high-quality sequences of Clitocybaceae species were retrieved and aligned with the newly generated sequences in this study that were checked and edited using Bioedit v7.0.9 [22]. Alignments were generated for single ITS, nrLSU, *rpb2*, and *tef1-α* datasets using MAFFT v7.313 [23]. Then, a four-locus combined dataset for Clitocybaceae, containing sequences of ITS, nrLSU, *rpb2*, and *tef1-α*, was generated by the PhyloSuite v1.2.1 software platform [24]. ModelFinder [25] was used for the selection of the best-fitting evolution model for the combined dataset. Maximum Likelihood (ML) analysis was performed with RAxML-8.2.10-WIN [26] with 1000 bootstrap replicates using the TIM2 model for the combined dataset. Bayesian Inference (BI) analysis was conducted with MrBayes v.3.2.6 [27] using the GTR+I+G model for the combined dataset, and the combined dataset was run for 8,000,000 generations. The best tree was viewed in FIGTREE v1.4.4 [28] and enhanced in Adobe Illustrator CC 2022.

## 3. Results

### 3.1. Phylogenetic Analyses

In the preliminary molecular analysis, *C. clavipes* sequences showed 96.4% similarity to *C. hunanensis* (NR_198326) with 98% query coverage for ITS, 99.4% similarity to *C. tibetica* (NG_243147) with 100% query coverage for nrLSU, 97.2% similarity to *Clitocybe dealbata* (DQ825407) with 100% query coverage for *rpb2*, and 80.5% similarity to *C. bisterigmata* (OP558049) with 97% query coverage for *tef1-α. Collybia carnea* sequences showed 97.8% similarity to *C. pannosa* (NR_198323) with 100% query coverage for ITS, 99.5% similarity to *C. phyllophila* (MK277723) with 99% query coverage for nrLSU, 96.2% similarity to *C. xylogena* (OP956582) with 92% query coverage for *rpb2*, and 94.6% similarity to *C. xylogena* (OP672233) with 60% query coverage for *tef1-α*. *Collybia violea* sequences showed 95.5% similarity to *C. nuda* (OP626948) with 100% query coverage for ITS, 98.7% similarity to *C. sordida* (OP646393) with 100% query coverage for nrLSU, 85.4% similarity to *C. nuda* (KJ136110) with 100% query coverage for *rpb2*, and 82.8% similarity to *C. nuda* (MG702630) with 100% query coverage for *tef1-α.*

**Figure 1:**
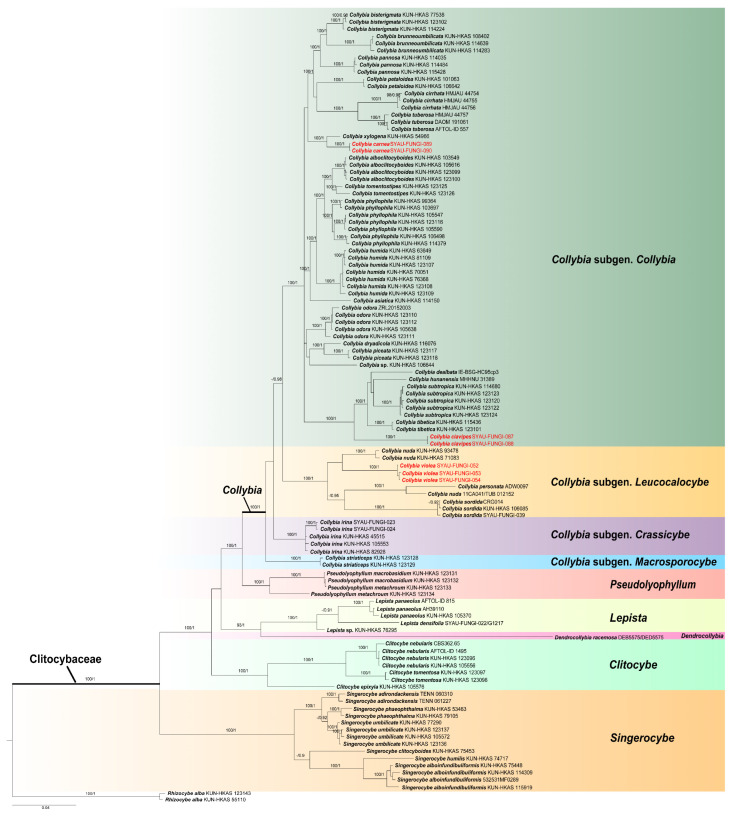
Phylogram of Clitocybaceae generated based on the combined ITS-nrLSU-*rpb2*-*tef1-α* dataset, with *Rhizocybe alba* as an outgroup. Bootstrap values (BP) ≥ 90% and Bayesian posterior probabilities (PP) ≥ 0.90 are shown around the branches. Newly generated sequences are shown in red.

In the phylogenetic analysis, this study analyzed 411 sequences (109 ITS, 108 nrLSU, 98 *rpb2*, 96 *tef1-α*) from 110 samples representing 44 species of Clitocybaceae. The combined dataset ITS-nrLSU-*rpb2-tef1-α* of Clitocybaceae comprised a total of 2792 positions. ML and BI resulted in almost identical tree topologies, and the BI tree was selected for display (Figure 1). The phylogenetic relationships of Clitocybaceae (Figure 1) were consistent with previous work [14].

The optimal topology indicates that Clitocybaceae represents a monophyletic lineage with high support (100% BP, 1.00 PP). As shown in the phylogenetic analyses of Clitocybaceae (Figure 1), a total of six clades can be recognized within the family as the following genera: *Collybia*, *Pseudolyophyllum*, *Lepista*, *Dendrocollybia*, *Singerocybe* and *Clitocybe*. This is in line with He et al. [14]. Sequences of *Collybia* species are divided into four clades, which is in line with He et al. [14]. In this present study, three new species, *C. clavipes, C. carnea* and *C. violea*, form a single clade with high support (100% BP, 1.00 PP). In detail, *C. clavipes* and *C. carnea* are located within the *Collybia* subgen. *Collybia*, and *C. violea* is located within the *Collybia* subgen. *Leucocalocybe*.

### 3.2. Taxonomy

**Figure 2:**
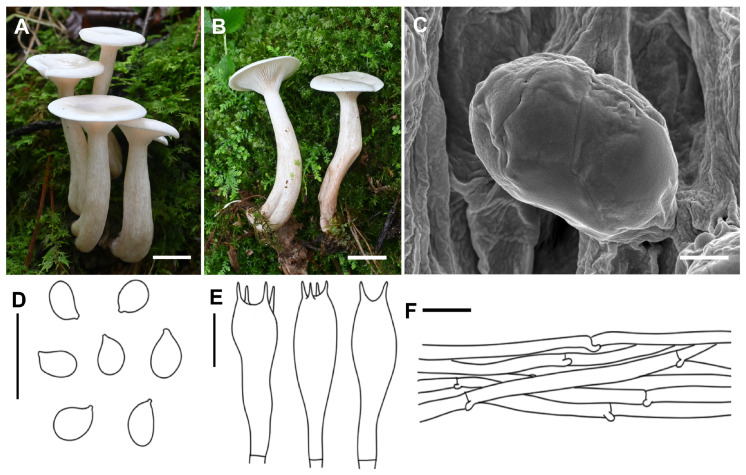
Morphological features of *Collybia clavipes* (SYAU-FUNGI-087, holotype). (**A**,**B**) Habitat and ba basidiomes; (**C**) SEM images of basidiospores. Line drawings of (**D**) basidiospores; (**E**) basidia; (**F**) pileipellis. Scale bar: (**A**,**B**) 1 cm; (**C**) 1 μm; (**D**–**F**) 10 μm.

***Collybia clavipes*** A.G. Xu & Y. Qi, sp. nov. (Figure 2)

*MycoBank*: MB858330

*Etymology*: “*clavipes*” refers to the clavate stipe.

*Diagnosis*: Differed from other known species of the subgenus by having small and white basidiomata, a clavate stipe and ellipsoid to sublacrymoid basidiospores.

*Holotype*: China, Xizang, Jilong County, in a mixed forest, 3104 m alt., 2 August 2023, A.G. Xu & Y. Qi (SYAU-FUNGI-087, holotype).

*Description*: Basidiomata small. Pileus 1.0–3.0 cm in width, applanate to slightly depressed at center; surface glabrous, white (1A1), hygrophanous; margin not striate, slightly inrolled. Lamellae decurrent, moderately crowded, 2–3 mm wide, white (1A1), with numerous tiers of lamellulae; edge entire or slightly eroded. Stipe 2.5–4.5 cm long × 0.5–1.2 cm wide, central, subclavate, hollow, gradually becoming enlarged downwards, sometimes slightly curved, surface white (1A1), brownish (6D4–8), longitudinally striate. Context thin, white.

Basidiospores (4.0) 5.0–6.0 (6.5) × 3.0–4.0 (4.5) μm, Q = 1.32–1.87, Qav = 1.64, ellipsoid to sublacrymoid, inamyloid, surface smooth under both the light microscope and electron microscope, not adherent, almost always single. Basidia 25.6–32.0 × 6.0–9.5 μm, clavate, four-spored, sterigmata up to 3.0–6.0 μm in length. Cystidia absent. Hymenophoral trama regular, hyphae cylindrical, hyphae up to 3.5–9.6 µm wide. Pileipellis hyphae cylindrical, hyphae up to 2.0–10.5 µm wide, with many branches. Stipitipellis hyphae cylindrical, hyphae up to 3.2–9.5 µm wide. Clamp connections present.

*Habitat*: Generally gregarious, more rarely solitary; saprotrophic on needle and broad leaf litter, in a mixed forest; summer.

*Distribution*: Only known from Xizang, China.

*Additional material studied*: China, Xizang, Jilong County, in a mixed forest, 3014 m alt., 20 August 2024, A.G. Xu & Y. Qi (SYAU-FUNGI-088).

*Notes*: Within *Collybia* subgen. *Collybia*, several species also have small and white basidiomes, such as *C. asiatica*, *C. bisterigmata* and *C. pannosa* [14]. However, the clavate stipe and ellipsoid to sublacrymoid basidiospores of *C. clavipes* make it easily distinguished from these species, which generally have a cylindrical stipe and non-sublacrymoid basidiospores. Furthermore, *C. asiatica* differs from *C. clavipes* by its infundibuliform basidiome and having basidiospores always adhering in tetrads [14]; *C. bisterigmata* differs by having two-spored basidia and larger basidiospores (6.0–7.5 (8.0) × 4.0–6.0 μm) [14]; *C. pannosa* differs by having felty pileus, basidiospores sometimes adhering in tetrads and irregularly arranged pileipellis [14].

**Figure 3:**
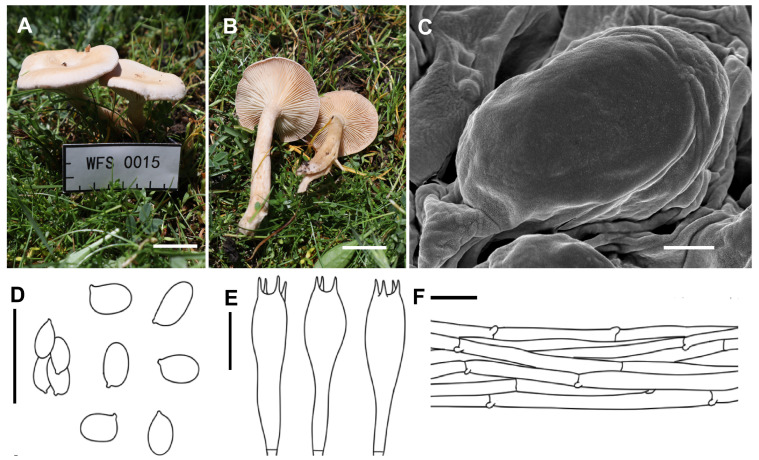
Morphological features of *Collybia carnea* (SYAU-FUNGI-089, holotype). (**A**,**B**) Habitat and ba basidiomes; (**C**) SEM images of basidiospores. Line drawings of (**D**) basidiospores; (**E**) basidia; (**F**) pileipellis. Scale bar: (**A**,**B**) 1 cm; (**C**) 1 μm; (**D**–**F**) 10 μm.

***Collybia carnea*** A.G. Xu & Y. Qi, sp. nov. (Figure 3)

*MycoBank*: MB858331

*Etymology*: “*carnea*” referring to a carneous basidiome.

*Diagnosis*: Differed from *C. dealbata* by having a carneous basidiome.

*Holotype*: China, Xizang, Dangxiong County, Ara wetland, in a mixed forest, 3104 m alt., 2 August 2024, A.G. Xu & Y. Qi (SYAU-FUNGI-089, holotype).

*Description*: Basidiomata small. Pileus 2.0–3.0 cm in width, plano-convex to nearly applanate, somewhat depressed at center; surface pinkish brown (5A2–3 to 6A2), becoming light brown (6B2–3) when old, white–pruinose, sometimes slightly lobate, not hygrophanous; margin not striate, inrolled, sometimes irregular, undulating. Lamellae adnate to subdecurrent, moderately crowded, thin, 1.0–2.0 mm wide, concolorous with pileus, with numerous tiers of lamellulae; edge entire. Stipe 4.0–5.0 cm long × 0.7–1.0 cm wide, central, cylindrical, hollow, sometimes slightly curved, concolorous with pileus, longitudinally striate, base not inflated, sometimes covered with whitish mycelium. Context about 1 cm thick, fleshy.

Basidiospores 4.5–6.0 × 2.5–4.0 (4.5) μm, Q = 1.30–2.02, Qav = 1.67, ellipsoid to elongate, inamyloid, surface smooth under both the light microscope and electron microscope, often single, rarely adherent. Basidia 25.5–31.0 × 6.2–8.6 μm, clavate, four-spored, hyaline, with sterigmata up to 2.0–6.5 μm in length. Cystidia absent. Hymenophoral trama regular, hyphae cylindrical, hyphae up to 3.5–9.6 µm wide. Pileipellis hyphae cylindrical, hyphae up to 3.0–9.5 µm wide, with many branches. Stipitipellis hyphae cylindrical, hyphae up to 3.0–8.5 µm wide. Clamp connections present.

*Habitat*: Scattered on rich soil among grasses, at the edge of a mixed forest, summer.

*Distribution*: Only known from Xizang, China.

*Additional material studied*: China, Xizang, Dangxiong County, on rich soil among grasses at the edge of a mixed forest, 3214 m alt., 20 August 2024, A.G. Xu & Y. Qi (SYAU-FUNGI-090).

*Notes*: Phylogenetically, *Collybia carnea* represents an independent clade and is a sister taxon to *C. xylogena* (Figure 1). Even so, *C. xylogena* can be easily distinguished by its brown to grayish brown pileus, relatively larger basidiospores (5.0–8.0 × 3–4.5 μm), and growing on wood [14]. Morphologically, *C. carnea* is closely related to *C. dealbata*, which also produces a small basidiome, a white–pruinose pileus with inrolled margin and medium-sized basidiospores, which are always mostly single [29,30]). However, *Collybia carnea* have a carneous basidiome, while *C. dealbata* have a white basidiome.

**Figure 4:**
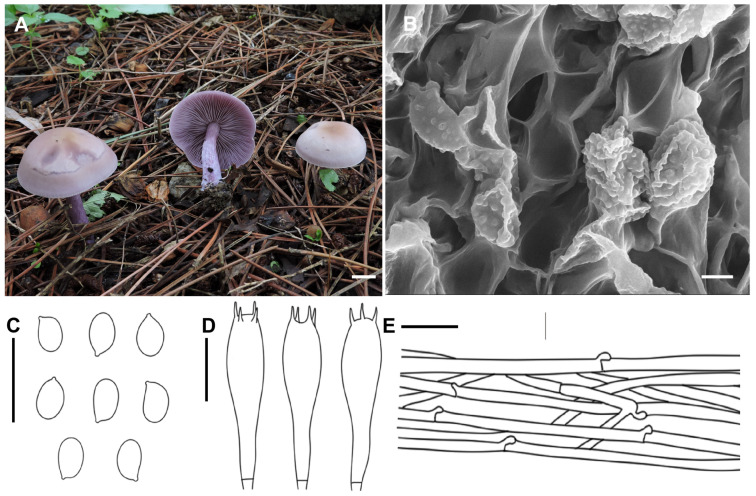
Morphological features of *Collybia violea* (SYAU-FUNGI-052, holotype). (**A**) Habitat and basidiomes; (**B**) SEM images of basidiospores. Line drawings of (**C**) basidiospores; (**D**) basidia; (**E**) pileipellis. Scale bar: (**A**) 1 cm; (**B**) 2 μm; (**C**–**E**) 10 μm.

***Collybia violea*** X.D. Yu & H.B. Guo, sp. nov. (Figure 4)

*MycoBank*: MB858332

*Etymology*: “*violea*” refers to violet basidiome.

*Diagnosis*: Distinguished from *C. sordida* and *C. nuda* by its slightly darker pileus color, and slightly smaller basidiospores.

*Holotype*: China, Liaoning Province, Shenyang City, Dongling Park, on rich soil among grasses, 177 m alt., 17 July 2019, X.D. Yu & H.B. Guo (SYAU-FUNGI-052, holotype).

*Description*: Basidiomata medium. Pileus 4.0–7.0 cm in width, at first convex, then gradually applanate, sometimes slightly depressed; surface lilac to grayish lilac (15 B2–3), becoming light brown (6C2–3) to ochraceous (6D5–8) from the center outwards; margin slightly inflexed when young, sometimes uplifted, irregular, undulating when mature. Lamellae adnexed to emarginate to subdecurrent, moderately crowded, thin, 2.0–3.0 mm wide, grayish violet to violet (17 B2–4), with numerous tiers of lamellulae; edge entire or slightly eroded. Stipe 2.5–5.0 cm long × 0.4–0.8 cm wide, central, cylindrical, solid or hollow, concolorous with lamellae, longitudinally striate, base slightly inflated. Context about moderately thick, fleshy.

Basidiospores 4.5–7.0 × 3.2–4.0 μm, Q = 1.30–1.82, Qav = 1.65, ellipsoid, inamyloid, surface smooth under the light microscope, finely verruculose under the electron microscope, not adherent, almost always single. Basidia 25.5–30.5 × 7.0–9.6 μm, clavate, four-spored, with sterigmata up to 3.0–5.5 μm in length. Cystidia absent. Hymenophoral trama regular, hyphae cylindrical, hyphae up to 3.0–9.2 µm wide. Pileipellis hyphae cylindrical, hyphae up to 2.5–9.5 µm wide, with many branches. Stipitipellis hyphae cylindrical, hyphae up to 2.0–8.5 µm wide. Clamp connections present.

*Habitat*: Saprophytic in small groups on rich soil among grasses, on roadsides.

*Distribution*: Known from northeastern China.

*Additional material studied*: China, Liaoning Province, Shenyang City, on the campus of Shenyang Agricultural University, on rich soil among grasses, 121 m alt., 31 August 2019, X.D. Yu & H.B. Guo (SYAU-FUNGI-053); China, Liaoning Province, Shenyang City, on the campus of Shenyang Agricultural University, on rich soil among grasses, 121 m alt., 31 August 2019, X.D. Yu & H.B. Guo (SYAU-FUNGI-054).

*Notes*: *Collybia sordida* and *C. nuda* could be easily mistaken for *C. violea* because they have lilac to grayish lilac basidiomata. *Collybia sordida* mainly differs by its hygrophanous and more dull-colored (pale bluish violet then pinkish beige with tinge olive at the center and in concentric zones on drying) pileus [31], and slightly larger basidiospores (7 × 4 μm [31]; 6–9 × 4–5 μm [32]). *C. nuda* can distinguish from *C. violea* by having much larger basidiomata (8–15 cm [31]; 2.5–10 cm [32]; 5–15 cm [33]) and slightly larger basidiospores (8 × 5 μm [31]; 5.5–9 × 4–5.5 μm [32]; 6.5–8.5 × 3.9–4.8 μm [33]).

## 4. Discussion

In the combined ITS-nrLSU-*rpb2*-*tef1-α* phylogenetic tree (Figure 1), Clitocybaceae forms a monophyletic clade, which is consistent with the previous study [14]. The three new species of *Collybia* described in this study occupy three independent positions, respectively. *Collybia clavipes* forms a distinct clade within the clade containing *C. dealbata*, *C. hunanensis*, *C. subtropica* and *C. tibetica*. *Collybia carnea* occupies a clade with the sister taxon, *C. xylogena*, but morphologically, *C. carnea* differs from *C. xylogena* by having a pinkish basidiome, smaller spores and growing on soil. *Collybia violea* forms a sister group with *C. nuda*, but morphologically, *C. violea* can be distinguished by having a smaller basidiome and smaller spores.

In this study, two new species belongs to the *Collybia* subgen. *Collybia*, which is a species-rich subgenus, with useful diagnostic features, such as basidiospore adhesion, two-spored or four-spored basidia, muscarine existence or non-existence, and growth substrates [14]. To some extent, this study further confirmed these diagnostic features’ usefulness to separate supraspecific taxa within the subgenus. For example, *C. clavipes* clusters with *C. subtropica*, *C. hunanensis*, *C. dealbata* and *C. tibetica* (Figure 1), all of which have four-spored basidia, basidiospores which are almost always single and grow on leaf litter or soil [14]. However, four-spored *C. carnea* has basidiospores which are always single and grow on soil, while two-spored *C. xylogena* has basidiospores which are often in tetrads and grow on rotten wood [14], although *C. carnea* clusters together with *C. xylogena* (Figure 1). This indicates that there may be still a number of new taxa remaining to be discovered within this clade.

*Collybia violea* belongs to *Collybia* subgen. *Leucocalocybe*. Due to interspecific similarities and phenotypic plasticity, *C. violea* has always been misidentified as *C. sordida* or *C. nuda.* However, our study finds there are distinct differences between *C. violea* and these species in both molecular data and morphological characteristics. Furthermore, previous phylogenetic analysis [14] based on worldwide ITS sequences of this subgenus suggested that there are five subclades of *C. nuda*. In the future, large amounts of related specimens need to be collected and detailed morphological studies need to be conducted to resolve the puzzles concerning which subclade could represent the true *C. nuda*.

## Figures and Tables

**Table 1:** Information on newly generated sequences in this study.

Species	Voucher Numbers	GenBank Accession Numbers				Location
		ITS	nrLSU	*rpb2*	*tef1-α*	
*Collybia clavipes*	SYAU-FUNGI-087	PQ594916	PQ594905	PQ801137	PQ801139	Xizang, China
	SYAU-FUNGI-088	PQ594917	PQ594906	PQ801138	PQ801140	Xizang, China
*C. carnea*	SYAU-FUNGI-089	PQ594919	PQ594908	PQ609669	PQ609675	Xizang, China
	SYAU-FUNGI-090	PQ594920	PQ594909	PQ609670	PQ609676	Xizang, China
*C. violea*	SYAU-FUNGI-052	MT482103	MT482097	PQ817427	MT510970	Liaoning, China
	SYAU-FUNGI-053	MT482104	MT482098	PQ817428	MT510971	Liaoning, China
	SYAU-FUNGI-054	MT482105	MT482099	PQ817429	MT510972	Liaoning, China

## Data Availability

All of the data that support the findings of this study are available in the main text.

## References

[1] Matheny P.B., Hofstetter V., Aime M.C., Moncalvo J.M., Ge Z.M., Yang Z.L., Slot J.C., Ammirati J.F., Baroni T.J., Bougher N.L. (2006). Major clades of Agaricales: A multilocus phylogenetic overview. Mycologia.

[2] Ammirati J.F., Parker A.D., Matheny P.B. (2007). *Cleistocybe*, a new genus of Agaricales. Mycoscience.

[3] Vizzini A., Musumeci E., Murat C. (2010). *Trichocybe*, a new genus for *Clitocybe puberula* (Agaricomycetes, Agaricales). Fungal Divers..

[4] Alvarado P., Moreno G., Vizzini A., Consiglio G., Manjón J.L., Setti L. (2015). *Atractosporocybe*, *Leucocybe* and *Rhizocybe: Three* new clitocyboid genera in the Tricholomatoid clade (Agaricales) with notes on *Clitocybe* and *Lepista*. Mycologia.

[5] Matheny P.B., Hughes K.W., Kalichman J., Lebeuf R. (2020). *Pulverulina*, a new genus of Agaricales for *Clitocybe ulmicola*. Southeast. Nat..

[6] He Z.M., Yang Z.L. (2021). A new clitocyboid genus *Spodocybe* and a new subfamily Cuphophylloideae in the family Hygrophoraceae (Agaricales). MycoKeys.

[7] Moncalvo J.M., Vilgalys R., Redhead S.A., Johnson J.E., James T.Y., Catherine Aime M., Hofstetter V., Verduin S.J., Larsson E., Baroni T.J. (2002). One hundred and seventeen clades of euagarics. Mol. Phylogenetics Evol..

[8] Walther G., Garnica S., Weiß M. (2005). The systematic relevance of conidiogenesis modes in the gilled Agaricales. Mycol. Res..

[9] Binder M., Larsson K.H., Matheny P.B., Hibbett D.S. (2010). Amylocorticiales ord. nov. and Jaapiales ord. nov.: Early diverging clades of agaricomycetidae dominated by corticioid forms. Mycologia.

[10] Sánchez-García M., Matheny P.B., Palfner G., Lodge D.J. (2014). Deconstructing the Tricholomataceae (Agaricales) and introduction of the new genera *Albomagister*, *Corneriella*, *Pogonoloma* and *Pseudotricholoma*. Taxonomy.

[11] Alvarado P., Moreau P.A., Sesli E., Khodja L.Y., Contu M., Vizzini A. (2018). Phylogenetic studies on *Bonomyces* (Tricholomatineae, Agaricales) and two new combinations from *Clitocybe*. Cryptogam. Mycol..

[12] Alvarado P., Moreau P.A., Dima B., Vizzini A., Consiglio G., Moreno G., Setti L., Kekki T., Huhtinen S., Liimatainen K. (2018). Pseudoclitocybaceae fam. nov. (Agaricales, Tricholomatineae), a new arrangement at family, genus and species level. Fungal Divers..

[13] He Z.M., Yang Z.L. (2022). The genera *Bonomyces*, *Harmajaea* and *Notholepista* from Northwestern China: Two new species and a new record. Mycol. Prog..

[14] He Z.M., Chen Z.H., Bau T., Wang G.S., Yang Z.L. (2023). Systematic arrangement within the family Clitocybaceae (Tricholomatineae, Agaricales): Phylogenetic and phylogenomic evidence, morphological data and muscarine-producing innovation. Fungal Divers..

[15] Qi Y., Yu X.D., Hou J.X., Guo H.B., Yang R.H., Xu A.G. (2024). Addition to the genus *Harmajaea* (Agaricales, Pseudoclitocybaceae): A new and a known species from China. Phytotaxa.

[16] Kornerup A., Wanscher J.H. (1963). Methuen Handbook of Colour.

[17] Doyle J.J., Doyle J.L. (1987). A rapid DNA isolation procedure for small quantities of fresh leaf tissue. Phytochem. Bull..

[18] White T.J., Bruns T., Lee S., Taylor J., Innes M.A., Gelfand D.H., Sninsky J.S., White T.J. (1990). Amplification and direct sequencing of fungal ribosomal RNA genes from phylogenetics. PCR Protocols: Methods and Applications.

[19] Vilgalys R., Hestsr M. (1990). Rapid genetic identification and mapping of enzymatically amplified ribosomal DNA from several cryptococcus species. J. Bacteriol..

[20] Matheny P.B. (2005). Improving phylogenetic inference of mushrooms with *RPB1* and *RPB2* nucleotide sequences *(Inocybe*; Agaricales). Mol. Phylogenetics Evol..

[21] Matheny P.B., Wang Z., Binder M., Curtis J.M., Lim Y.W., Nilsson H., Hughes K.W., Hofstetter V., Ammirati J.F., Schoch C.L. (2007). Contributions of *rpb2* and *tef1* to the phylogeny of mushrooms and allies (Basidiomycota, Fungi). Mol. Phylogenetics Evol..

[22] Hall T.A. (1999). BioEdit: A user-friendly biological sequence alignment editor and analysis program for Windows 95/98/NT. Nucleic Acids Symp. Ser..

[23] Katoh K., Standley D.M. (2013). MAFFT multiple sequence alignment software version 7: Improvements in performance and usability. Mol. Biol. Evol..

[24] Zhang D., Gao F., Jakovlić I., Zou H., Zhang J., Li W.X., Wang G.T. (2020). PhyloSuite: An integrated and scalable desktop platform for streamlined molecular sequence data management and evolutionary phylogenetics studies. Mol. Ecol. Resour..

[25] Kalyaanamoorthy S., Minh B.Q., Wong T.K.F., Haeseler A.V., Jermiin L.S. (2017). ModelFinder: Fast model selection for accurate phylogenetic estimates. Nat. Methods.

[26] Stamatakis A. (2014). RAxML version 8: A tool for phylogenetic analysis and post-analysis of large phylogenies. Bioinformatics.

[27] Ronquist F., Teslenko M., van der Mark P., Ayres D.L., Darling A., Höhna S., Larget B., Liu L., Suchard M.A., Huelsenbeck J.P. (2012). MrBayes 3.2: Efficient Bayesian phylogenetic inference and model choice across a large model space. Syst. Biol..

[28] Rambaut A. FigTree v1.4.4: Tree Figure Drawing Tool. https://github.com/rambaut/figtree/releases.

[29] Harmaja H. (1969). The genus *Clitocybe* (Agaricales) in Fennoscandia. Karstenia.

[30] Bigelow H.E. (1982). North American species of Clitocybe.

[31] Bon M. (1987). The Mushrooms and Toadstools of Britain and North-Western Europe.

[32] Breitenbach J., Kränzlin F. (1991). Fungi of Switzerland, Vol. 3-Boletes and Agarics.

[33] Buczacki S. (2014). Collins Fungi Guide: The Most Complete Field Guide to the Mushrooms and Toadstools of Britain & Europe.

